# How can we get sober from the influence of the alcohol industry?

**DOI:** 10.1016/S2214-109X(22)00024-9

**Published:** 2022-02-01

**Authors:** Maristela G Monteiro, Zila M Sanchez

**Affiliations:** a Department of Noncommunicable Diseases and Mental Health, Pan American Health Organization, Washington, DC 20037, USA; b Department of Preventive Medicine, Universidade Federal de São Paulo, São Paulo, Brazil

The Article by Pepita Barlow and colleagues in *The Lancet Global Health*^1^ is an important piece of scientific evidence that shows once again the influence and interference of the alcohol industry in areas where their profits can be affected. In the Article, the authors carefully established that the same tactics and strategies of the tobacco industry to influence and interfere with policy making have been used by the alcohol industry for the past decade at World Trade Organization (WTO) discussions on alcohol health warning labelling, compromising the implementation of potentially the most cost-effective measures to reduce alcohol consumption and related harms worldwide. The well collected and analysed data brings robust evidence to the field of alcohol policy and advances knowledge by exposing, for the first time, that the WTO is another international forum permeated by alcohol industry influence. The core finding was that 117 (55·2%) of 212 statements made by member representatives of the WTO Technical Barriers to Trade (TBT) Committee replicated the well known and outworn arguments of the alcohol industry against evidence-based public policy.^2^ The discourses act to deny science by downplaying the extensive harmful effects of alcohol use. The evidence that WTO is another forum for alcohol industry influence suggests that WHO should not only work with governments but all UN organisations to minimise the negative effects of vested interests to protect public health, to achieve a unified position on placing health and equity at the centre of development.

The methodology used was an original way to analyse discussions held at the WTO TBT discussions, utilising all public records of the WTO discussions on alcohol products in the past decade, which are not easily accessible otherwise. However, despite the clear indication that WTO member statements reflected similar positions among the alcohol industry, it remained unclear as to how these positions are adopted by governments, and whether they are a result of direct or indirect influence of economic operators. The descriptions of the influence and economic power of these large corporations, often more powerful and influential than many governments, must lead us to reflect on the actions that are needed by public health officials, advocates, and researchers to globally protect society, as previously outlined by several alcohol policy experts.^3^ Rapid and extensive effort is needed to inform the public and policy makers on how existing alcohol policies are going backwards, meaning that alcohol consumption and harms will increase, probably to levels higher than those before the COVID-19 pandemic. Such changes to alcohol policies come at a time when businesses are struggling and asking for incentives to be kept open. Thus, the arguments against alcohol health policies are appealing to the public, who have also struggled with job losses and increased health-care expenses. Furthermore, it is now more difficult to show why loosening alcohol policies to increase sales is a bad economic decision. If before the pandemic the costs of alcohol consumption were already several times higher than the profits from sales,^4^ promoting even more sales can only be detrimental to national economies and health systems, at a time when governments are spending so much in health care already to manage COVID-19. Doing more harm to increase profits is illogical, when the profits will not be able to pay for the harm done. On the contrary, alcohol taxes should increase to enable governments to build health systems that are more resilient. However, when the alcohol industry is able to influence governments and institutions, as shown by Barlow and colleagues,^1^ economic strength will tend to prevail over global health protection.

The authors conclude their paper by mentioning the need for transparency regarding the vested interests of the alcohol industry. However, the situation calls for more impactful measures than just a transparent declaration of conflicts of interest. Transparency alone does not mitigate the harmful impact of this influence on public health if the voice of alcohol industry is given the same credibility as those of other non-state actors, and commercial interests prevail over public health. Therefore, the views and inputs from actors associated with the alcohol industry should be clearly separated from the alcohol policy decision process in all fora. Even indirect participation, as found by the authors, should be prevented.

The key message from the paper, along with the real-world implications, is that key stakeholders in public health need to stop the ability of the alcohol industry to influence and interfere with policy decision making. Governments can unite to develop binding regulations for alcohol corporations, and clear rules to prevent vested interests having an influence on the development, adoption, and implementation of alcohol policies to reduce alcohol consumption and related harms. Although alcohol health warning labels are only a small part of public health measures needed to protect consumers from alcohol-related risks, it should be unacceptable to have health warnings that serve only those who cause harms and blame the individual for their choices.

We declare no competing interests. The views expressed in this Comment are those of the authors only and do not necessarily reflect the position or views of the Pan American Health Organization.
